# Electrical Microstimulation of Visual Cerebral Cortex Elevates Psychophysical Detection Thresholds

**DOI:** 10.1523/ENEURO.0311-18.2018

**Published:** 2018-10-30

**Authors:** Jackson J. Cone, Amy M. Ni, Kaushik Ghose, John H. R. Maunsell

**Affiliations:** 1 Department of Neurobiology, Biological Sciences Division, University of Chicago, Chicago, IL 60637; 2 Department of Neuroscience and Center for the Neural Basis of Cognition, University of Pittsburgh, Pittsburgh, PA 15260; 3 Seven Bridges Genomics, Cambridge, MA 02142

**Keywords:** microstimulation, perception, prosthesis, psychophysics, vision

## Abstract

Sensory prostheses can restore aspects of natural sensation by delivering electrical current directly into sensory circuits. An effective sensory prosthetic should be capable of generating reliable real-time perceptual signals for hours each day over many years. However, we still know little regarding the stability of percepts produced by electrical microstimulation of cerebral sensory cortex when stimulation is delivered repeatedly over long periods. Developing methods that yield highly sensitive and reliable assessments of a subject’s sensitivity to stimulation is important for developing prosthetic devices that can mimic the constant stream of information inherent in daily experience. Here, we trained rhesus monkeys to report electrical microstimulation of their primary visual cortex (V1) and measured how repeated stimulation affected the minimal electrical current needed to generate a percept (behavioral detection threshold). Using adaptive staircase procedures with a two-alternative forced-choice (2AFC) detection task, we obtained highly reliable detection threshold measures with as few as 100 trials. Using either chronically implanted or acutely inserted microelectrodes, we found that repeated electrical microstimulation elevated detection thresholds, with effects persisting between daily testing sessions. Our results demonstrate task designs that can support rapid and reliable measurements of detection thresholds, and point to the need for validation that detection thresholds in targeted structures will be sufficiently stable in the face of the amount of chronic stimulation that will be required for effective sensory prosthetics.

## Significance Statement

Delivering electrical current into sensory brain areas could enable those with compromised sensory systems to partially recover lost senses. Whether repeated stimulation of central sites in the brain changes the ability of the stimulated site to support perception remains unresolved. We present methods for rapid, bias-free and repeatable measures of behavioral thresholds for detecting microstimulation and show that repeated electrical stimulation of visual cortex impairs the ability of monkeys to perceive that stimulation. The results have important implications for the development and use of sensory prosthetics.

## Introduction

Neural prostheses hold great promise for individuals with sensory loss caused by trauma, disease, or genetic predisposition. Direct application of electrical current to sensory structures in the brain can produce robust percepts by activating neurons that have been otherwise deprived of sensory input. Natural sensations depend on the integration of constantly varying streams of information arising from thousands of parallel channels. Considerable progress has been made in increasing the number of electrodes that can be used simultaneously for stimulation, with the goal of approaching densities consistent with natural sensory pathways. However, another key goal for neural prosthetics is to generate percepts for many hours a day, stably over years or decades. This need poses challenges related to power delivery, biocompatibility and electrical currents that are appropriately limited to avoid compromising the underlying neural tissue. Moreover, there are important questions regarding how chronic artificial activation alters the responses of neuronal circuits.

Electrical stimulation applied through neurostimulators used for clinical deep brain stimulation (DBS) can be therapeutic for many years. Nevertheless, the efficacy of DBS often changes over time. For example, when DBS is used in dystonia patients, positive effects often emerge gradually over weeks to months (Ruge et al., 2011) and more frequent stimulator adjustments are required with DBS for dystonia than with DBS for Parkinson’s disease or essential tremor (Butson et al., 2006). This suggests that sustained electrical stimulation can reorganize neuronal circuits, possibly to varying degrees in different structures. However, DBS might not provide precise insights for sensory prosthetics. The mechanisms of action for DBS are not well understood (Herrington et al., 2016) and DBS electrodes are relatively large and deliver currents that are high (Butson et al., 2006) compared with those typically used when stimulating sensory structures with microelectrodes (Bak et al., 1990; Schmidt et al., 1996; Bradley et al., 2005). Moreover, DBS electrodes are often placed in brain regions with circuit architectures that differ considerably from that of sensory cortices.

Microelectrode studies have similarly suggested that sustained stimulation can reorganize neuronal circuits (Ni and Maunsell, 2010). Microstimulation of primary visual cortex (V1) in monkeys has revealed that repeated stimulation can rapidly reduce an animal’s ability to detect that stimulation (Bartlett et al., 1977; Torab et al., 2011). Other studies that have measured how electrical microstimulation thresholds are influenced by repeated stimulation have shown that detection thresholds for stimulation of sensory cortex can be stable, or even improve, over periods longer than a year (Normann et al., 1999; Rousche and Normann, 1999; Callier et al., 2015). However, these studies measured detection thresholds at long intervals without continuous stimulation in between, a condition that does not closely correspond to expected microelectrode use in a sensory prosthetic. A recent study did deliver electrical microstimulation for 20 h a week across several months (Rajan et al., 2015). The authors found no histopathology associated with prolonged stimulation, however they did not measure the effects of repeated stimulation on detection thresholds.

Progress toward a practical sensory prosthetic will require a thorough characterization of the long-term stability of percepts produced by electrical microstimulation that is sustained over long periods. Given the many factors that can affect behavioral measurements and the widely ranging, sometimes contradictory, observations that have come from microstimulation studies to date, this effort could be advanced by using testing methods that yield highly sensitive and repeatable measures of behavioral thresholds for detecting microstimulation. Here, we lay a foundation for such studies by measuring thresholds for detecting microstimulation of V1 in macaque monkeys with methods that allow for rapid, bias-free estimates. We show that these methods offer enough precision to detect threshold elevations resulting from stimulating individual cortical sites for as little as 30 s.

## Materials and Methods

Other findings based on the data described here have been reported previously (Ni and Maunsell, 2010; Ghose and Maunsell, 2012). All animal procedures were in accordance with the Institutional Animal Care and Use Committees of Baylor College of Medicine or Harvard Medical School.

### Behavioral task

**Figure 1.**
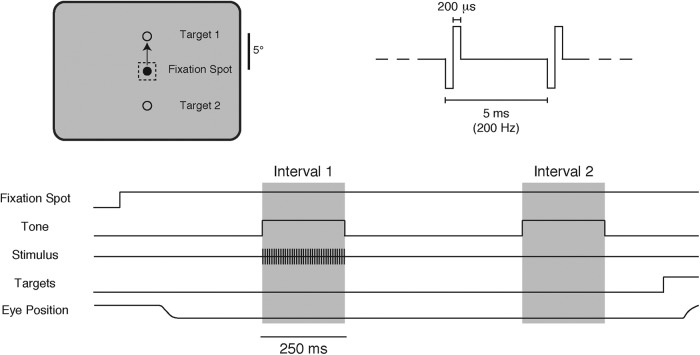
2AFC task. During fixation, electrical microstimulation was delivered during one of two 250-ms time intervals that were marked by auditory tones and separated by 500 ms. Two response targets appeared 250 ms after the end of the second interval and the animals indicated which interval contained the stimulus by making a direct saccade to the appropriate target (target 1 for interval 1, target 2 for interval 2). The electrical stimulus was a 250 ms, 200-Hz train of biphasic constant-current pulses, anodal phase first, with each phase lasting 200 µs. Thresholds were determined by using different current levels on different trials using an adaptive staircase procedure.

We trained four adult male rhesus monkeys (*Macaca mulatta*) to perform a two-alternative forced-choice (2AFC) detection task (Fig. 1). Each animal had scheduled access to water and earned juice rewards by reporting which interval contained the stimulus in each trial. Initially, the monkeys learned to report the appearance of a small visual stimulus. Each trial began with the appearance of a small fixation spot in the center of a video display with a gray background (12 cd/m^2^). After the animal had fixed its gaze on this spot, two 250-ms intervals occurred in sequence, each accompanied by a tone and separated from one another by a 500-ms gap. During one interval, randomly selected for each trial, a small, two-dimensional white Gaussian stimulus appeared at an eccentric location on the display. Following a 250-ms delay after the end of the second interval, two target spots appeared, 5° above and below the fixation point. The animal indicated which interval contained the stimulus by making a saccade directly to the appropriate target: the target above the fixation spot for interval 1 or the target below for interval 2. The animal only needed to report the interval in which the stimulus occurred, and not its location or other qualities.

On each trial, the contrast of the visual stimulus was assigned one of a fixed set of values that spanned the animal’s detection threshold. The location of the stimulus was moved regularly, but remained in each position for at least a few hundred trials. All parameters related to the behavioral task (e.g., eye position, visual stimuli, microstimulation, reward delivery, etc.) and on-line displays of behavioral performance were controlled using custom software.

Once performance with visual stimulation became stable, the visual stimulus was removed and replaced with electrical stimulation of a V1 site through a microelectrode. The electrical stimulus was a 250 ms, 200 Hz train of biphasic constant-current pulses, anodal phase first, with each phase lasting 200 µs. The currents delivered were limited so that they never exceeded 50 μA (amplitude of an individual phase). Detection thresholds for microstimulation were determined using an adaptive staircase procedure (see Data analysis).

### Surgical procedures

Each monkey was implanted with a titanium head post and a scleral search coil under general anesthesia. After training on the behavioral task, monkeys were surgically prepared for electrical microstimulation. Two monkeys were implanted with a 6 × 8 platinum microelectrode array (Utah arrays, Blackrock Microsystems; 0.2–1.5 MΩ impedance at 1 kHz; Maynard et al., 1997) in V1 of each hemisphere. The microelectrode array consisted of 1 mm long electrodes arranged in a 6 × 8 rectangular grid with a 400-μm pitch. Before each stimulation session, the arrays were connected to a constant current stimulator using a percutaneous connector. Only one microelectrode in the array was stimulated at a time for detection threshold measurements. Two other monkeys were implanted with cylinders over V1. Access to cortex was achieved through small (2–6 mm) craniotomies that were made inside the cylinder under anesthesia. The dura mater remained intact. Before acute microstimulation sessions, a custom-built glass-coated Pt/Ir microelectrode (0.2–1.5 MΩ impedance at 1 kHz) was advanced transdurally each day into the opercular region of V1.

### Data analysis

All data were analyzed using MATLAB (The MathWorks, Inc.). We used QUEST (Watson and Pelli, 1983) for adaptive staircase measurements. Behavioral mean hit rates were fit to a Weibull function and threshold was taken as the contrast needed to reach 63% of the span from chance to saturating performance (∼82% correct). One hundred behavioral responses were used for threshold measurements with the adaptive staircase procedure.

To determine the rate of threshold elevation with chronically implanted electrodes, we calculated threshold change normalized by the number of trials between pairs of threshold measurements. To avoid effects of changing motivation within experimental sessions, only measurements collected on different days were compared. To ensure that all change measures were independent, each threshold value was used for only a single measure. Except for these two constraints, pairs were assigned at random, with confidence intervals estimated using a bootstrap procedure.

## Results

The experimental design was optimized to produce sensitive and reliable measures of behavioral thresholds. Several aspects were taken into consideration. First, V1 is an ideal location for investigating microstimulation induced percepts because work with human subjects has shown that microstimulation of a site in V1 can reliably evoke the sensation of a small spot of light in a corresponding retinotopic location (a phosphene; Brindley and Lewin, 1968; Dobelle and Mladejovsky, 1974; Schmidt et al., 1996; Lewis et al., 2016; Bosking et al., 2017). Percepts are less reliably evoked in later stages of visual cortex (Murphey et al., 2009). Second, the monkeys were trained to perform a 2AFC task because this task avoids arbitrary threshold elevations resulting from subjects adopting a conservative criterion for reporting whether a stimulus occurred, as can occur with yes/no designs (Green and Swets, 1966). Third, we used an adaptive psychometric procedure to estimate thresholds as efficiently as possible (Watson and Pelli, 1983).

Once each animal was proficient at the detection task using the visual stimulus, we replaced the visual stimulus with electrical microstimulation of V1. Although the transition to microstimulation was abrupt, each animal rapidly transferred to reporting detection of electrical stimulation of V1. In some cases, this transfer was immediate, and in all cases, animals were reliably reporting V1 electrical stimulation within a few days. Once animals were familiar with responding to microstimulation, we never failed to obtain a behavioral threshold of <50 µA from any of the over 250 V1 sites that we tested.

### Thresholds measured in the 2AFC task are unaffected by small response biases

Because the stimulus was equally likely to appear in either interval, a bias toward reporting one interval would impair performance and elevate threshold estimates. None of our animals had a strong interval bias. Individually, monkeys 1–4 selected interval 1 on 48.4%, 50.2%, 56.1%, and 55.2% of all trials. A feature of the 2AFC task is that it is highly tolerant of small biases like these, and the effect of interval biases on detection estimates in 2AFC tasks can be assessed analytically (Green and Swets, 1966; p 408). The largest of the four biases would have caused hit rates to be underestimated by ∼0.1%. Overall, the effects of interval bias were negligible compared to other factors affecting threshold estimates, such as systematic changes in motivation, which we discuss below.

### Microstimulation detection thresholds rise within and across days

**Figure 2.**
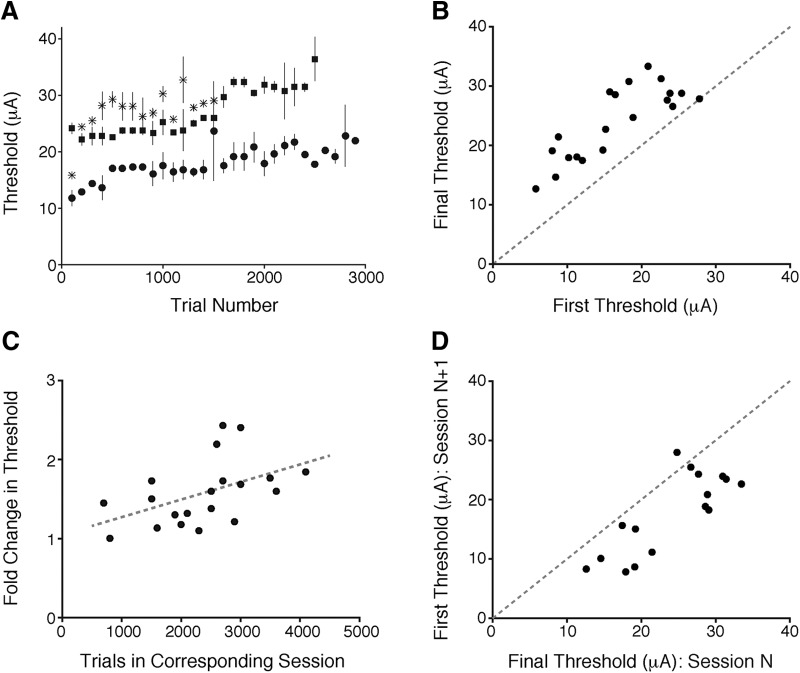
Detection thresholds rise within sessions and recover partially between sessions. ***A***, Example single session data from three representative microelectrodes showing that detection thresholds rise when a given site is repeatedly microstimulated during the daily session. Monkey 1: circles and squares; monkey 2: asterisks. Error bars = 95% CI. ***B***, Detection thresholds consistently rise across a session. For each session (two electrode sites per animal; monkey 1: 15 total sessions; monkey 2: five total sessions), the rise in threshold across trials was fitted with an exponential function. Individual points represent the initial (*x*-axis) and final (*y*-axis) detection threshold measurements from the fitted data. ***C***, The rise in detection threshold within a session is correlated with the number of stimulated trials in that session (*p* < 0.05), showing that the change in threshold increases with increasing stimulation. ***D***, Thresholds partially recover between consecutive days of electrical microstimulation. Individual points represent the final threshold estimate from one session (*x*-axis) and first threshold estimate obtained during the next session (*y*-axis).

We examined the stability of detection thresholds by repeatedly measuring detection thresholds for electrical currents delivered through chronically implanted microelectrodes in two monkeys. Behavioral thresholds consistently rose over the course of repeated measures. Figure 2*A* plots changes in behavioral thresholds associated with repeated microstimulation through three representative microelectrodes. Each symbol plots thresholds for one microelectrode that was used repeatedly for 15–30 100-trial threshold measurements over the course of 1 d’s testing. Detection thresholds typically increased gradually when a V1 microelectrode site was repeatedly stimulated throughout the course of a daily session.

To quantify the elevation in detection threshold within daily sessions, we fit threshold data from each day with an exponential function, and used this fit to determine an initial and final threshold for each day. Figure 2*B* plots the fit for the first threshold measurement on a given day against the fit for the final threshold measurement for that day. In almost all cases the final threshold was elevated. To determine whether the change in detection threshold depended on the amount of stimulation within a daily session, we examined the relationship between the change in threshold and the number of stimulation trials for that electrode in the corresponding session. There was a significant positive relationship between the change in threshold in a session and the number of stimulation trials in the corresponding session (*p* = 0.036, Pearson’s *r* = 0.47; Fig. 2*C*), showing that changes in detection threshold were proportional to the amount of stimulation.

**Figure 3.**
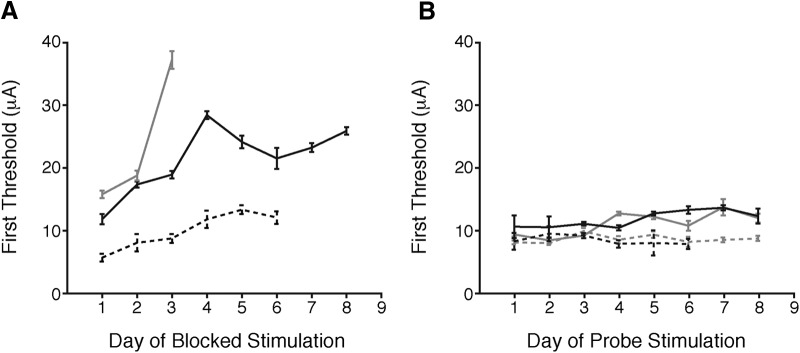
Detection thresholds rise across days of repeated stimulation of the same cortical site. ***A***, Detection thresholds at the start of a daily behavioral session increase across repeated days of stimulation. Lines depict the first detection threshold estimate made per day across successive days of electrical microstimulation (monkey 1: black lines; monkey 2: gray line). Error bars represent 95% CI. ***B***, Detection thresholds remain stable at four nearby electrode sites in monkey 1 where only one threshold estimate was made each day.

There was a partial recovery of the detection thresholds between daily sessions. Figure 2*D* plots the threshold measured at the end of a session against the first threshold measured using the same microelectrode during the subsequent session. In almost every case, thresholds were lower after a long period (∼20 h) without stimulation. However, this recovery was typically incomplete. Figure 3*A* shows behavioral thresholds measured using stimulation through three different microelectrodes over 3–8 d across which each microelectrode was stimulated repeatedly. In each case, the lowest threshold was obtained on the first day of testing, and thresholds rose over successive days. Thus, repeated electrical microstimulation of cerebral cortex can reduce behavioral sensitivity both within and across days, with elevated thresholds persisting after overnight periods with no stimulation. While fluctuations in motivation over the course of a session could contribute to within-day threshold changes, effects that persist across days must depend on other processes.

To quantify the accumulating threshold elevation across days, for each microelectrode site we randomly selected pairs of threshold measurements and computed the change in threshold between them, normalized by the number of trials intervening between measurements (see Materials and Methods). To eliminate effects of increasing satiety within daily sessions, we constrained each pair such that each threshold came from a different day. We found a significant positive rate of threshold change across sessions (mean = 1.038-fold increase, or 3.8%, per 1000 trials; 1.034–1.042 95% CI; bootstrap; average of 6100 trials between measurements; range: 600–20,800 trials).

The threshold elevations seen in Figures 2, 3*A* occurred when each microelectrode was tested many times within each session (single session range: ∼600–4000 trials of stimulation or ∼1200–28,000 nC). However, the data in Figure 2*C* suggest that thresholds rise less when stimulation is limited. To examine this, we used four other microelectrodes to make a single behavioral threshold measurement each day (100 trials of stimulation, or ∼200 nC). These sparsely stimulated sites showed little evidence of elevation of detection threshold across days (Fig. 3*B*). We did a linear regression to determine whether there was a significant rise in threshold across days of probe stimulation. Only one of the four sites had a significantly positive slope (solid gray line; *p* < 0.01 for slope parameter). These observations confirm that across-day threshold elevation depends on the amount of stimulation.

### Elevated detection thresholds with acutely inserted electrodes

The above results demonstrate detection thresholds for V1 rise following repeated microstimulation through chronically implanted microelectrodes. While chronic implants are the most relevant for efforts to develop cortical microstimulation prosthetics, we wanted to see whether threshold elevation depended on having a device chronically implanted on the overlying cortical surface. We therefore did additional experiments in two other monkeys in which individual transdural microelectrodes were inserted and removed each day. In these experiments, two 100-trial threshold determinations were made at each V1 site with the same adaptive staircase procedure used for chronically implanted microelectrodes (see Materials and Methods). The electrode was then advanced to a new site, with successive sites separated by at least 100 µm. We examined whether the threshold from the second measurement at each site was elevated relative to that from the first.

**Figure 4.**
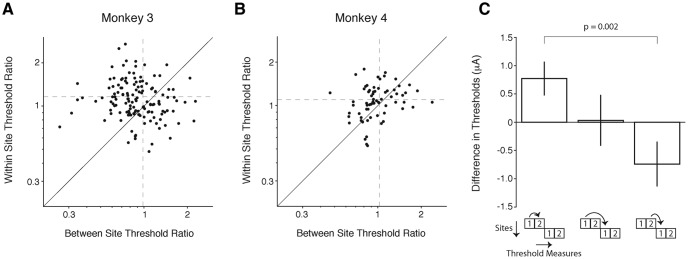
Detection thresholds increase over 100 trials of stimulation. Two 100-trial threshold measurements were made at each V1 site. ***A***, ***B***, Each point represents the ratio of threshold measurements between two subsequent measurements when the electrode was advanced between measurements (*x*-axis) compared to when both measurements were made at the same site (*y*-axis; ***A***: monkey 3, 140 sessions; ***B***: monkey 4, 69 sessions). Dashed lines indicate mean *x*,*y* values. The ratios of thresholds within site were significantly greater than those between sites (both animals: *p* < 0.05). ***C***, Bar plot shows the mean difference (±1 SEM) between pairs of threshold measurements. Repeated stimulation of the same site elevated thresholds for the second measurement compared to the first (left bar). This measure corresponds the *y*-axis ratios in ***A***, ***B***. After two threshold measurements, the microelectrode was advanced by 100 μm into a new site. The difference in thresholds between the first measurement at a new site and the first measurement at the previous site was not significantly different from zero (middle bar), indicating that there was no systematic change in threshold across a session for previously unstimulated sites. Consistent with these observations, the difference between the first threshold measurement at a new site and the second threshold measurement at the previous site was negative (right bar), reflecting lower thresholds for unstimulated cortex relative to cortex that has been previously stimulated. This measure corresponds to the *x*-axis ratios in ***A***, ***B***.

For both monkeys, the second threshold estimate was on average ∼7% higher than the first (monkey 3: first threshold 11.7 μA, 0.6 SEM; second threshold 12.6 μA, 0.5 SEM; *t*_(175)_ = -2.75; *p* = 0.0065; monkey 4: first threshold 14.8 μA, 0.8 SEM; second threshold 15.9 μA, 0.9 SEM; *t*_(92)_ = -3.15; *p* = 0.0022; paired *t* tests). Thus, the threshold to detect microstimulation of V1 increases measurably over as few as 100 trials of microstimulation. However, subjects’ motivation can gradually wane during a daily session, and we wanted to confirm that these changes in threshold did not arise from uncontrolled changes in effort between successive measures. For this, we compared the ratio of threshold measurements for two sequentially collected measurements at the same site (within site) with the ratio of thresholds between the second measurement at one site and the first measurement at the next site (between sites). The threshold ratios were significantly greater within site compared to between sites for both monkeys (Fig. 4*A*,*B*; monkey 3: mean within-site = 1.15, 0.03 SEM; mean between sites = 0.098, 0.04 SEM; monkey 4: mean within site = 1.10, 0.03 SEM; mean between sites = 1.02, 0.04 SEM; both *p* < 0.05, Wilcoxon signed-rank test). Figure 4*C* shows the difference in thresholds for different comparisons within and between sites for both monkeys. Only within site differences were reliably positive (left bar; mean within site difference = +0.77 μA, 0.3 SEM), again supporting that thresholds elevate following repeated stimulation of the same cortical site. In contrast, comparing the first threshold measurements at successive sites yielded a difference that was near zero (middle bar, mean = +0.03 μA, 0.5 SEM), indicating there was no systematic relationship between threshold measurements across previously unstimulated sites throughout a session. Correspondingly, the difference between the first measurement at a new site and the second measurement at the previous site was reliably negative (right bar, mean = -0.74 μA, 0.4 SEM), reflecting the elevation in threshold observed at a repeatedly stimulated site relative to the first threshold measurement at a new site. Statistical tests revealed that these differences in thresholds were significantly different (*p* < 0.001; Kruskal–Wallis test), and *post hoc* analyses revealed that within site differences (left bar) were significantly greater than successive measurements made between sites (right bar; *p* = 0.002). Taken together, these data suggest that there was little consistent increase in threshold at successive sites within a session when the electrode was advanced frequently. Moreover, this supports the view that the threshold elevations seen with repeated stimulation through chronically implanted microelectrodes depends on electrical stimulation rather than factors such as satiety, fatigue, or distractibility.

The rate of threshold elevation with transdural electrodes was substantially greater than the within-session rate seen with chronically implanted electrodes (a factor of 1.14 per 100 trials, 0.03 SEM; Fig. 4*A*,*B*; compared with a factor of 1.038 for chronic electrodes over 1000 trials). The slower rate of change with chronically implanted electrodes was likely due to detection thresholds rising at an ever-slower rate with repeated stimulation at the same site. For chronically implanted electrodes, the average rate of change estimated from the first half of sessions was greater than the average rate of change measured during the second half [first half: 1.042-fold increase (4.2%) per 1000 trials, 1.037–1.047 95% CI; second half: 1.033-fold increase (3.3%) per 1000 trials, 1.027–1.037 95% CI; *p* = 0.01].

## Discussion

To gain insight into how repeated electrical microstimulation of sensory cortex alters the ability of the stimulated site to support perception, we trained monkeys to do a task that allowed us to precisely and rapidly calculate the amount of current needed to produce a behaviorally detectable V1 activation. With this approach, we were able to measure behavioral thresholds that were highly consistent across days with limited stimulation (Fig. 3*B*), as well as thresholds that changed rapidly with repeated stimulation (Figs. 2*A*, 4*A*,*B*).

Our results suggest that careful consideration of task design will be critical for measuring the performance of neural prosthetics. For example, while we obtained behavioral thresholds below 50 µA from every site we tested in V1, a similar study that also used Utah arrays to stimulate sites in monkey V1 was unable to measure thresholds from 74 of 82 microelectrodes despite using currents of up to 92 µA (Torab et al., 2011). Multiple factors are likely to have contributed to this difference. We believe the primary causes are that the experiments in the other study included: a yes/no task design, which allows subjects to adopt a conservative response criterion; thresholds based on few behavioral responses (sometimes only 20); and rewarding near-threshold responses randomly.

Our approach showed that thresholds rose slightly, but steadily, with repeated stimulation. Multiple days of repeated stimulation led to persistent threshold elevation that recovered only partially between daily stimulation sessions. In contrast to our findings, other studies have reported that behavioral thresholds can remain stable over long periods. Callier et al. (2015) showed that microstimulation thresholds in primate somatosensory cortex can be stable over week to months. Rousche and Normann (1999) stimulated through Utah arrays in cat auditory cortex and found stable detection thresholds for up to 100 d. However, in both cases threshold measurements were made only at widely-spaced intervals within those long testing periods, a situation in which we similarly found little threshold elevation (Fig. 3*B*).

We previously showed that the detection of electrical microstimulation can improve gradually with practice over thousands of trials (Ni and Maunsell, 2010). In that investigation, electrical microstimulation was delivered through acutely inserted electrodes that were regularly advanced between threshold measurements, such that no individual cortical site was stimulated for an extended period. It seems likely that the processes supporting such threshold improvements are distinct from those that underlie the threshold elevations described here. There is no reason to doubt that both occur when a single site is chronically stimulated; however, the threshold elevations that come from chronic stimulation of a given site are larger and faster than the threshold improvements that have been seen when chronic stimulation is avoided.

Earlier studies have similarly reported that repeated electrical microstimulation of cortical sites increases detection thresholds (Bartlett et al., 1977; Torab et al., 2011), though these studies did not monitor thresholds over long periods of ongoing microstimulation. Davis et al. (2012) stimulated monkey V1 using Utah arrays and found significant threshold elevations even when tests were widely spaced (an average of five measurements spanning an average of 125 d). To our knowledge, detection thresholds for cortical stimulation have not been monitored over long periods of ongoing microstimulation, a condition of foremost relevance for sensory prostheses.

The cause of threshold elevations is unknown. It is unlikely that microstimulation of this sort causes any gross damage. Rajan et al. (2015) used Utah arrays in monkey somatosensory cortex to deliver electrical microstimulation for 4 h/d for six months. They found no differences in gliosis or loss of neuronal density near stimulated electrode tips compared with unstimulated electrodes, using stimulation intensities of up to 100 µA. While they did not measure detection thresholds in their study, their results suggest that the changes in detection threshold we observed were due to short- and long-term neuronal adaptations to microstimulation and not due to mechanically or electrically induced damage at the electrode tips.

It is possible that the loss of behavioral sensitivity arises from chemical reactions at the electrode surface. Metals differ greatly in their susceptibility to hydrolysis, and while platinum and iridium have excellent characteristics in this regard (White and Gross, 1974), it is possible that electrodes made of materials other than those we used would not lead to threshold elevations. For example, electrodes coated in a sputtered iridium oxide film (SIROF; Cogan et al., 2004) have higher damage thresholds compared to other iridium coatings (Negi et al., 2010). Appropriate electrode metals or coating might allow unlimited stimulation without raising thresholds. Nevertheless, validation of threshold stability with parameters matched to expected patterns of stimulation would be needed.

The threshold elevation we described might represent a neurobiological response of sensory neurons to chronic stimulation that is independent of the physiochemical properties of the electrode. Various brain structures might differ in their susceptibility to such effects. In particular, chronic stimulation through cochlear implants is highly effective and stable over decades of use (Lenarz et al., 2012), with issues of instability focusing on topics such as physical movements caused by bone growth in very young patients (Roland et al., 1998). The retina (Ghezzi, 2015) or thalamus (Pezaris and Eskandar, 2009) might have more stable responses to chronic microstimulation than cerebral cortex. If different structures respond differently to long-term electrical stimulation, validation will need to be repeated for proposed prosthetic target sites. Behavioral testing in such validation might be widely spaced as long as appropriate ongoing stimulation was applied to approximate the expected use of a prosthetic.

Prosthetic technologies hold great promise for rescuing lost sensation. The ideal sensory prosthetic will be capable of generating real-time artificial percepts for many hours a day throughout years or decades of continuous use. Consequently, it is critical to understand the stability of the relationship between stimulation and perception over a life cycle of normal use. The useful life of a sensory prosthetic could depend as much on the stability of its perceptual effects when engaged in daily stimulation as it does on factors like gross biocompatibility of materials and mean time between component failures.

## Synthesis

Reviewing Editor: Li Li, New York University Shanghai Decisions are customarily a result of the Reviewing Editor and the peer reviewers coming together and discussing their recommendations until a consensus is reached. When revisions are invited, a fact-based synthesis statement explaining their decision and outlining what is needed to prepare a revision will be listed below.

In this study, the authors used a new design of microstimulation detection paradigm and found repetitive microstimulation gradually increased the stimulation detection threshold within and across recording sessions. Such results have implications for those chronically implanted microstimulation for sensory prosthetics. Overall, the results are robust according to their elaborate analysis, but more details need to be clarified.

1. The author claimed that their design allows for accurate estimation of microstimulation detection threshold without bias. But through the whole text, the author didn't provide any evidence that subjects show no choice preference during the final measurement period. Could the author provide the situation about bias (PSE) in addition to the detection threshold for all the measurements? For example, the authors show that the detection threshold was increasing over time due to repetitive stimulation, then what about the bias? Is the bias correlated with the detection threshold measured?

2. Other researchers have found that microstimulation in macaque sensory cortices could be detected with low magnitude (e.g. Ni and Maunsell, 2010), and the threshold was decreasing over trials/time. After enough training, the detection threshold could be reduced to as low as a few microA! The authors here reported an opposite effect: increasing threshold over trials/time. The authors need to discuss about possible reasons that may explain different results from these previous studies.

3. Before each stimulation session, did the authors measure the response property (e.g. location of the receptive field) of the V1 site they applied microstimulation? Was the property stable for each day? Did the response property change before and after stimulation?

4. Results (Paragraph 7): What does ‘nC’ mean?

5. Figure 3B. The upper limit of y axis is far higher than the maximum value of the dataset. If setting the y limit property, it seems that the black line and the grey line do increase a little bit. Is this trend significant?

6. The sentence “The change in threshold within sites was significantly greater than the change in threshold between sites.....” has no direct indication from Figure 4C-D. Adding (or change C&D into) one figure about the comparison of threshold change between and within sites with statistics is a better presentation.

7. I feel the biggest possibility of the increasing detection threshold is due to the interaction of the tissue around the electrode tip. If you put an electrode in the same place and apply microstimulation repetitively, the efficiency of the stimulation would be gradually diminished because the electrode tip is gradually corrupted, or the cells are damaged. Could the author surely exclude this possibility? In this sense, the acute penetration experiment could be useful, and the authors showed that the detection threshold was also increased, although with a lower magnitude, compared to the first site. However, it suffered that it may be due to the sensory properties have been changed compared the first site. So related to point #3, when using acute electrode penetrations, did the response property of the consecutive stimulated sites differ a lot from that for the first site?
